# A comparative analysis of Y chromosome and mtDNA phylogenies of the *Hylobates* gibbons

**DOI:** 10.1186/1471-2148-12-150

**Published:** 2012-08-21

**Authors:** Yi-Chiao Chan, Christian Roos, Miho Inoue-Murayama, Eiji Inoue, Chih-Chin Shih, Linda Vigilant

**Affiliations:** 1 Department of Primatology, Max-Planck Institute for Evolutionary Anthropology, Deutscher Platz 6, Leipzig, 04103, Germany; 2 Gene Bank of Primates and Primate Genetics Laboratory, German Primate Center, Kellnerweg 4, Göttingen, 37077, Germany; 3 Wildlife Research Center, Kyoto University, 2–24 Tanaka-Sekiden-cho Sakyo-ku, Kyoto, 606-820, Japan; 4 Graduate School of Science, Kyoto University, Kitashirakawa Oiwake-cho, Sakyo-ku, Kyoto, 606-8502, Japan; 5 Animal Division, Taipei Zoo, No.30 Sec.2 Xinguang Rd, Taipei City, 11656, Taiwan

**Keywords:** Y chromosome phylogeny, Phylogenetic relationships, Divergence times, Mitochondrial genome, Gene flow

## Abstract

**Background:**

The evolutionary relationships of closely related species have long been of interest to biologists since these species experienced different evolutionary processes in a relatively short period of time. Comparison of phylogenies inferred from DNA sequences with differing inheritance patterns, such as mitochondrial, autosomal, and X and Y chromosomal loci, can provide more comprehensive inferences of the evolutionary histories of species. Gibbons, especially the genus *Hylobates*, are particularly intriguing as they consist of multiple closely related species which emerged rapidly and live in close geographic proximity. Our current understanding of relationships among *Hylobates* species is largely based on data from the maternally-inherited mitochondrial DNAs (mtDNAs).

**Results:**

To infer the paternal histories of gibbon taxa, we sequenced multiple Y chromosomal loci from 26 gibbons representing 10 species. As expected, we find levels of sequence variation some five times lower than observed for the mitochondrial genome (mtgenome). Although our Y chromosome phylogenetic tree shows relatively low resolution compared to the mtgenome tree, our results are consistent with the monophyly of gibbon genera suggested by the mtgenome tree. In a comparison of the molecular dating of divergences and on the branching patterns of phylogeny trees between mtgenome and Y chromosome data, we found: 1) the inferred divergence estimates were more recent for the Y chromosome than for the mtgenome, 2) the species *H. lar* and *H. pileatus* are monophyletic in the mtgenome phylogeny, respectively, but a *H. pileatus* individual falls into the *H. lar* Y chromosome clade.

**Conclusions:**

Based on the ~6.4 kb of Y chromosomal DNA sequence data generated for each of the 26 individuals in this study, we provide molecular inferences on gibbon and particularly on *Hylobates* evolution complementary to those from mtDNA data. Overall, our results illustrate the utility of comparative studies of loci with different inheritance patterns for investigating potential sex specific processes on the evolutionary histories of closely related taxa, and emphasize the need for further sampling of gibbons of known provenance.

## Background

DNA sequences have often been used to investigate the evolutionary relationships of populations and species [1]. Phylogenetic trees are reconstructed using DNA sequence data to illustrate the evolutionary relationships of species, and the extent of evolutionary changes (e.g. number of substitutions) recorded in DNA sequences can be used to infer the timing of divergence events. However, it can be difficult to use DNA sequence data to confidently resolve the evolutionary relationships between recently diverged taxa, because too little time may have elapsed for the accumulation of sufficient genetic differences to provide phylogenetic resolution among taxa [2]. Also, particularly in cases in which population or taxon divergences have occurred over a short space of time (i.e. short branch lengths in the species tree), incomplete lineage sorting may cause the patterns of their molecular divergences to be inconsistent with the actual patterns of organism divergences [3]. Mitochondrial DNA (mtDNA), as a uniparentally inherited genomic region, has higher mutation rates, as well as a smaller effective population size, than typical autosomal loci and hence has a shorter coalescence time conducive to resolving phylogenetic relationships of recently diverging species [4,5]. Short segments of the mitochondrial genome (mtgenome) have long been used for phylogenetic reconstruction, and moreover sequencing of the entire mtgenome has been adopted to provide improved resolution for reconstructing robust phylogenies of species with recent rapid evolution and to facilitate the molecular dating of divergence events within their phylogenies (e.g. [6-9]). However, due to the maternal inheritance of mitochondria, the phylogenetic relationships inferred through mtgenomes, intrinsically, reveal matrilineal evolutionary history only. Hence, although less commonly used, as a paternally inherited counterpart of mtDNA, genetic data from the non-recombining portion of Y chromosome (NRY) loci are good candidates for extracting evolutionary information of the patrilineal history for mammals, including primates, and in comparison with data from the mtDNA, reveal potentially contrasting patterns of male and female phylogeny and gene flow [10-17].

**Figure 1:**
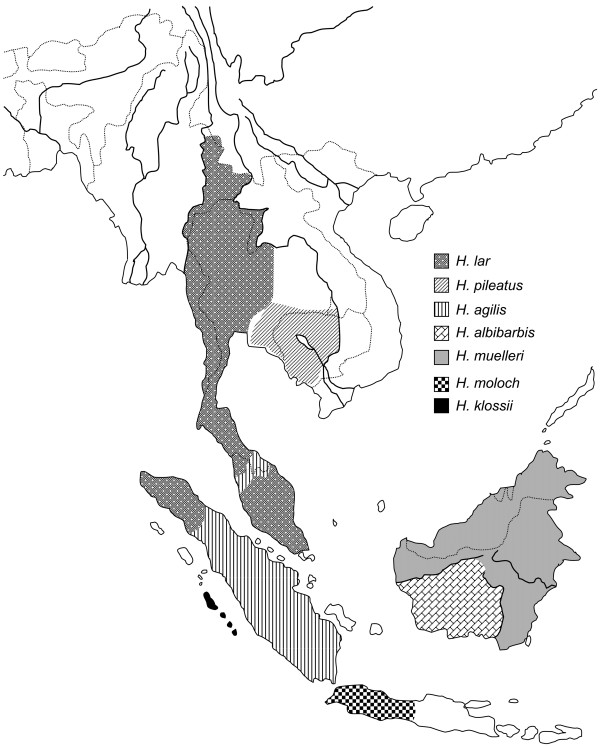
**Approximate geographic distribution of*Hylobates*gibbons.** Dotted and solid lines indicate country borders and major rivers, respectively. Adapted from Thinh et al. [20].

While all of the modern great apes currently exist as distinct, geographically-discontinuous species, gibbons are unique among the hominoids in consisting of multiple closely-related species living now or recently in close geographic proximity to one another. Gibbons are described as 14–19 nominal species divided into four genera: *HylobatesHoolockNomascus*, and *Symphalangus*, [18-21]. Of the four genera, *Hoolock* and *Symphalangus* contain just two and one species, respectively, while *Hylobates* comprises some half-dozen species as does *Nomascus*. The *Hylobates* gibbons are of particular interest as they apparently underwent a rapid speciation process and successfully colonized not only parts of mainland Southeast Asia but also the Sundaland (Figure 1) and are thus the most widespread group of gibbons [6,20,22]. The systematics and evolutionary relationship of the *Hylobates* members have been investigated using morphological characters, vocal traits of the songs, analysis of chromosomal morphology and DNA sequence data (e.g. [6,20,23-26]). In particular, the increasing number of DNA sequence studies have enabled molecular inferences on several aspects of the *Hylobates* evolutionary history, such as phylogenetic relationships, divergence time estimation and possible routes of dispersal [6,20,26-31]. The sequence data have demonstrated that *Hylobates* species are monophyletic [6,20,30,31] and suggested that their radiation began around 4 million years ago (mya) [6,20,26]. However, the phylogenetic relationships among *Hylobates* species had remained unresolved based on DNA sequences of short mitochondrial segments [20,28-31], the combined sequence of mtDNA, Y-linked and X-linked loci [26] or nuclear sequences from autosomes and X chromosomes [32] until a recent work depicting a well-supported *Hylobates* phylogeny based on more than 15 kb-length DNA sequences of the mitochondrial genome [6].

Although genetic studies have thus been conducive to the understanding of the *Hylobates* evolutionary relationships, most of the analyses in these studies have primarily relied upon mtDNA sequence data [6,20,27-30]; in other words, the most current molecular inferences are based solely upon the matrilineal evolutionary history of the *Hylobates*. Therefore, sequence data from Y-linked loci of gibbons would provide an opportunity to compare the evolutionary histories of both sexes, as differences arising either by chance assortment of genetic loci or arising out of different patterns of sex-mediated gene flow may be reflected by different branching patterns observed in the mtDNA and Y chromosome phylogeny trees. Thus far, phylogenetic information of gibbon Y chromosomes has been limited to short segments (800–2630 bp in length) from a few loci (SMCY, UTY, and ZFY) [26,33,34]. In the present study we used information from studies of Y chromosome variation in primates or other mammals [33,35-38], to generate sequences of multiple NRY loci in gibbons. Our sampling of 26 male individuals includes three of the four extant genera and 10 gibbon species, including six *Hylobates* species. Based on a large amount of gibbon Y chromosomal sequence data (> 165 kb in total), we reconstruct the gibbon Y chromosome phylogeny and compare these results with those inferred from various mtDNA data as well as with inferences from the previous mtgenome sequencing of the same individuals [6].

## Results

### 454 Sequencing and nucleotide diversity levels of Y chromosome loci

We used 454 sequencing technology to sequence seven PCR amplicons from six Y chromosome loci for each of 26 gibbons. A total of 229,599 raw reads were generated and the 56.8% of these reads with an average PHRED-equivalent base quality score of 30 were used for subsequent individual barcode sorting and contig assembly. In summary, for each individual, we obtained an average of 4,481 assembled reads with an average length of 214 bp per read, thus yielding approximately 1 Mb of sequence data corresponding to 146-fold coverage. The coverage for each individual ranged from 88- to 222-fold. After contig assembly, the consensus sequences of each amplicon were generated and each of the 26 gibbon individuals had seven amplicon sequences. We then generated the datasets for each amplicon as well as the concatenated dataset in which seven amplicon sequences were combined, representing a total length of ~6.4 kb. Multiple sequence alignments were carried out for all datasets.

By calculating two nucleotide diversity indices (π and θ_w_), we found that the diversity levels varied among the seven Y chromosomal amplicons (Table 1): the SMCY and TSPY showed relatively high nucleotide diversity indices, whereas the lowest diversity level was found in the amplicon UTY. We further calculated the nucleotide diversity of the concatenated datasets for each genus and each *Hylobates* species containing multiple sampled individuals, respectively (Table 2) and compared the Y chromosome sequence variation observed here to that of the mtgenome sequences from the same number of individuals. We calculated two nucleotide diversity indices π and θ_w_ for the 26 published mtgenome sequences [6]. We found that, overall, the values for the gibbon Y chromosomes were more than five times lower than that of the mtgenomes (Table 2). This lower nucleotide diversity level for the Y chromosome was also observed consistently in the three sampled genera as well as in the four multiply sampled *Hylobates* species.

**Table 1:** Nucleotide diversity indices of Y chromosomal amplicons in 26 gibbons

	**Sites analyzed (bp)**	**S**	**π%**	**θ**_**w**_**%**
Amplicons				
DAZ-1	1,524	62	0.923	1.006
DAZ-2	1,273	49	0.896	1.009
DBY	714	33	1.257	1.211
RPS4Y	567	23	0.916	1.063
SMCY	403	23	1.340	1.496
TSPY	725	41	1.477	1.482
UTY	875	23	0.574	0.689

S is the number of segregating sites; π is nucleotide diversity [77]; θ_w_ is estimated from S [78].

**Table 2:** Nucleotide diversity indices of Y chromosomal loci and mtDNAs in gibbons

**Sequence**	**Genus or species**	**N**	**Sites analyzed (bp)**	**S**	**π%**	**θ**_**w**_**%**
Y loci	All	26 (10)	6081	254	1.000	1.095
	*Hylobates*	19 (6)	6102	118	0.427	0.553
	*Nomascus*	4 (3)	6198	24	0.231	0.221
	*Symphalangus*	3 (1)	6192	2	0.022	0.022
	*H. agilis*	3	6111	13	0.142	0.142
	*H. lar*	10	6108	5	0.016	0.029
	*H. moloch*	2	6123	2	0.033	0.033
	*H. pileatus*	2	6109	32	0.524	0.524
Mtgenome^a^	All	26 (10)	11846	2570	6.022	5.685
	*Hylobates*	19 (6)	12970	1899	3.985	4.189
	*Nomascus*	4 (3)	14095	606	2.358	2.345
	*Symphalangus*	3 (1)	15325	91	0.396	0.396
	*H. agilis*	3	15187	367	1.613	1.611
	*H. lar*	10	14111	147	0.310	0.368
	*H. moloch*	2	15414	108	0.701	0.701
	*H. pileatus*	2	15374	307	2.000	2.000
Cytochrome b^b^	All	85 (16)	1140	429	9.030	7.505
	*Hoolock*	5 (2)	1140	40	1.930	1.684
	*Hylobates*	39 (7)	1140	256	5.392	5.311
	*Nomascus*	37 (6)	1140	184	4.128	3.866
	*Symphalangus*	4 (1)	1140	23	1.140	1.100

N is the number of individuals and in parentheses is the number of species sampled for each genus; S is the number of segregating sites; π is nucleotide diversity [77]; θ_w_ is estimated from S [78]. ^a^Calculations were carried out using mtgenome sequences excluding the control regions from the 26 individuals [6]. ^b^Calculations were carried out using 85 cytochrome b gene sequences (GenBank IDs of the cytochrome b genes were listed in [20]).

Among the three sampled genera, we found that the genus *Hylobates* showed the highest diversity levels for its mtgenome and Y chromosome DNA sequences. However, the differing sample sizes for the various genera and species in our study, and especially the larger sample size for the genus *Hylobates*, renders it difficult to explicitly compare the diversity indices among genera. Therefore, we randomly resampled three species from the six *Hylobates* species represented here and then randomly resampled one individual from each of the resampled species, respectively, to obtain similar sample sizes among the three genera. We then calculated two nucleotide diversity indices for the three resampled *Hylobates* individuals and repeated this resampling procedure 10 times. We obtained: π = 0.415-0.748% and θ_w_ = 0.415-0.742% for Y chromosome and π = 4.955-5.819% and θ_w_ = 4.929-5.862% for mtgenome, respectively and so we still observed the highest level of nucleotide diversity for *Hylobates*. The lack of readily available samples for the *Hoolock* genus and our limited number of samples of *Nomascus* relative to *Hylobates* make it inappropriate for us to attempt statistical tests or draw strong conclusions concerning comparisons of diversity among gibbon genera. In addition, among the four *Hylobates* species, *H. pileatus* showed the highest level of nucleotide diversity in both of Y chromosome and mtgenome sequences.

### Phylogenetic analyses

Multiple alignments with outgroups were conducted for each Y chromosomal amplicon and the proportions of variable sites varied among amplicons (11.33%-21.65%; Table 3). The highest proportion of variable sites were observed in the TSPY dataset (160/739 = 21.65%), which was almost twice that of the UTY dataset (103/909 = 11.33%). We reconstructed non-partitioned maximum-likelihood (ML) and Bayesian inference (BI) majority-rule trees for each amplicon dataset and found that the branching patterns varied from amplicon to amplicon (Additional file 1). Among these trees, we observed that both ML and BI trees based on two amplicons, namely the SMCY and TSPY, had relatively better resolution for depicting the relationships of three sampled genera albeit with weak support values (Additional file 1). In contrast, the sequence data of the other five amplicons were unable to provide information on the phylogenetic relationship of the three genera. Despite the low resolution of the trees of seven individual amplicons considered singly, the monophyly of the genera *Hylobates*, *Nomascus* and *Symphalangus* were well supported in all trees, except those of RPS4Y and UTY, in which *Nomascus* and *Symphalangus* but not *Hylobates* were recognized as monophyletic taxa. Given these results, it is not surprising that we also found inconsistent phylogenetic relationships of *Hylobates* species among the individual amplicon trees which lacked good statistical support values on the nodes (Additional file 1).

**Table 3:** Length and sequence variation of 7 Y chromosomal amplicons from 26 gibbons

**Datasets**	**Alignment size (bp)**	**Invariable sites**	**Variable sites**	**Parsimony-informative sites**
DAZ-1	1,715	1,234	257	123
DAZ-2	1,337	1,052	182	74
DBY	744	590	96	44
RPS4Y	572	468	95	41
SMCY	424	320	77	39
TSPY	739	560	160	73
UTY	909	753	103	39
Concatenated	6,440	4,977	970	433

**Figure 2:**
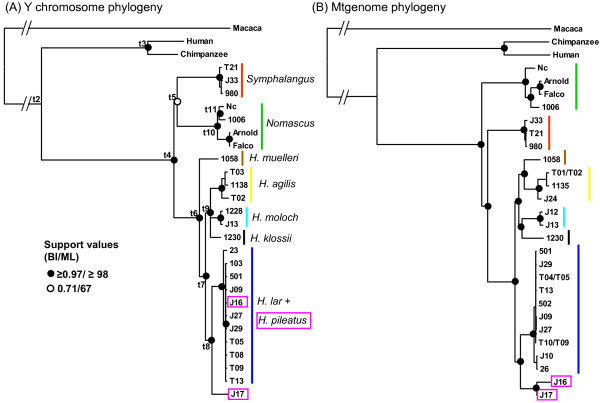
**Bayesian majority-rule trees of gibbon phylogenies.** The trees were constructed using the sequences from the partitioned concatenated dataset of seven Y chromosome amplicons (A) and the mtgenome sequences excluding the control regions (B), in which the T01/T02, T04/T05 and T10/T09 indicate the mother-son relationship of these individual pairs. The support values for the nodes according to the Bayesian inference posterior probability (BI) and maximum likelihood bootstrap (ML) are shown as the circles (filled circles: ≥0.97 BI, ≥ 98 ML; open circles: 0.71 BI, 67 ML). The two *H. pileatus* individuals are boxed. In A, nodes labelled t1-t11 are discussed in the text and shown in Table 4.

Since all of the NRY loci are linked, we concatenated the sequences of the seven amplicons to obtain single sequences for each of the 26 gibbons as well as the outgroups. We aligned these concatenated sequences and reconstructed phylogenetic trees using partitioned ML and BI analyses. The alignment was 6,440 bp in length and 15.1% of the sites were variable while 6.7% were parsimony-informative (Table 3). We found topologically identical majority-rule trees from the ML and BI analyses with similar support values (Figure 2). The monophyly of each genus received high support (BI posterior probability: 1.00 and ML bootstrap: 100). Two of the three sampled genera (*Nomascus* and *Symphalangus*) formed a weakly supported clade (BI: 0.71 and ML: 67). Within the genus *Nomascus*, *N. concolor* was closer to *N. leucogenys* than to *N. gabriellae*. Within the genus *Hylobates*, sequences from *H. muelleri* were basal to others with high support values followed by two groups in which representatives of the species *H. lar* and *H. pileatus* separate from an unresolved trichotomy consisting of members of the three remaining species ( *H. agilis*, *H. moloch* and *H. klossii*). Interestingly, sequences from *H. lar* and *H. pileatus* are not reciprocally monophyletic but occur in a single clade (BI: 1.00 and ML: 100).

The mtgenome phylogeny tree was also reconstructed based on the 26 retrieved sequences in which most sampled individuals were the same or the direct maternal relatives of those used in the Y chromosome tree (Figure 2, also see Methods for details). We obtained identical topologies from the ML and BI analyses, showing no basal taxon and all well-resolved nodes (BI: 1.00 and ML: ≥ 99) in the *Hylobates* mtgenome phylogeny. The species *H. lar* and *H. pileatus* are reciprocally monophyletic to each other. The *Hylobates* phylogenetic relationships inferred from this subset of mtgenome sequences were entirely consistent with the previous highly supported mtgenome tree which featured a larger number of individuals [6].

### Estimation of divergence times

We used the concatenated Y chromosome sequence dataset and implemented a Bayesian MCMC approach with the relaxed clock model in the program BEAST to estimate divergence times in the gibbon Y chromosome phylogeny. Beyond giving two monophyletic groups (*Macaca*-apes and human-chimpanzee) as the two fossil calibration points, we did not constrain the phylogeny with an *a priori* fixed topology. The maximum-clade-credibility tree generated by the BEAST analysis showed the same topology as produced in the ML and BI trees, respectively. The estimates of divergence times with 95% highest posterior densities (HPD) suggest that the initial split within gibbons occurred 5.21 mya (Table 4). The Y chromosome sequences of *Hylobates* spp. diverged at about 2.56 mya, followed by a split between two species groups, the clade ( *H. lar* +  *H. pileatus*) and the trichotomy ( *H. agilis*/ *H. moloch*/ *H. klossii*) at 2.04 mya. The estimated time to most common ancestor (TMRCA) of Y chromosomes for the clade ( *H. lar* +  *H. pileatus*) was 1.55 mya and the TMRCA of the trichotomy ( *H. agilis/H. moloch/H. klossii*) was 1.45 mya. Moreover, we inferred a recent divergence at approximately 0.6 mya of Y chromosomes between *N. concolor* and *N. leucogenys* (node t11).

**Table 4:** Bayesian estimates of divergence times inferred from the concatenated dataset of seven Y chromosomal amplicons

**Node**	**Divergence**	**Mean**	**95% HPD**
t1*	*Macaca*-apes	29.51	25.21-33.73
t2	gibbons-human/chimpanzee	16.88	13.74-20.05
t3*	human-chimpanzee	5.40	4.45-6.30
t4	gibbons	5.21	4.01-6.44
t5	*Nomascus*- *Symphalangus*	4.87	3.76-6.06
t6	*Hylobates*	2.56	1.90-3.27
t7	2 groups of *Hylobates* spp.	2.04	1.49-2.61
t8	*H. lar* + *H. pileatus*	1.55	1.06-2.08
t9	*H. agilis*/ *H. moloch*/ *H. klossii*	1.45	0.99-1.91
t10	*Nomascus*	0.84	0.50-1.25
t11	*N. concolor*- *N. leucogenys*	0.60	0.28-0.93

The estimated divergence dates are in million years before present. MRCA denotes the most recent common ancestor. HPD, highest posterior density

*Nodes used for calibration.

## Discussion

### Nucleotide diversity of gibbon Y chromosomes

Based on sequence data of Y chromosome loci examined here, we found that the sequence diversity of the examined segments of the Y chromosome was at least five-fold lower than that of the mtgenome (Table 2). This lower nucleotide diversity of Y chromosome sequences relative to mtDNA diversity is not specific to gibbons but appears to be a common pattern in mammals [39-46]. The large difference in diversity between Y chromosome and mtDNA is probably due to the much higher substitution rate of mtDNA compared to nuclear DNA causing a high rate of evolution in mitochondria [47], as well as strong selection on sex-limited chromosomes (e.g. background selection and selective sweeps) that could contribute to the relatively low sequence variability observed on mammalian Y chromosomes [48].

We further examined nucleotide diversity levels for each of the three sampled genera. In spite of the limited sample sizes, we found that *Hylobates* apparently has a higher level of nucleotide diversity than *Nomascus* and *Symphalangus* for both the mtgenome as well as the Y-chromosome (Table 2). This inference is supported by a recent examination of the mtDNA cytochrome b sequences using fairly comprehensive sampling of extant gibbon taxa [20] which provides estimates consistent with those based on our mtgenome and Y chromosome datasets of relatively small sample size (Table 2). Moreover, their data with the inclusion of sampling of *Hoolock* species showed the highest π and θ_w_ for genus *Hylobates* (Table 2) and suggested that *Hylobates* may be the most genetically diverse gibbon genus.

Owing to the limited samplings of the six species and lack of samples from the *H. albibarbis* species, we were unable to extensively compare nucleotide diversity levels among *Hylobates* species. However, we found that *H. pileatus* showed 1.25 times the nucleotide diversity levels of *H. agilis* on the mtgenome but exhibited nearly four times the nucleotide diversity of *H. agilis* on the Y chromosome. This relatively high Y chromosome diversity in *H. pileatus* was consistent with its paraphyletic Y chromosome lineage, in which the sequences derived from two different lineages (Figure 2 and Table 2). While our results provide some indication of the relative levels of sequence diversity present in the various gibbon taxa, we caution that comparisons of diversity must be considered provisional until the adoption of widespread sampling and analysis of individuals of known geographic provenance.

### Dating divergences in gibbon evolutionary history

Different molecular inferences of divergence times may be derived from the paternally-inherited Y-chromosome and the maternally-inherited mtDNA, and such differences may reflect the different evolutionary histories of maternal and paternal lineages as well as simply the influence of chance on the coalescent history of these two single loci. We find that the molecular divergence times inferred from the two loci are largely consistent with broad confidence intervals, but that the coalescence times inferred from the mtDNA data tend to be older than those from the Y-chromosome. Specifically, the divergences of Y chromosomes among the three sampled gibbon genera began around 5.2 (4.0-6.4) mya (Table 4), in comparison to the 8.7 (5.3-12.5) mya [6] or the 8.0 (6.6-9.7) mya [22] inferred from mtgenome data from these same genera (*HylobatesNomascus* and *Symphalangus*). Within the genus *Hylobates*, the first divergence of *Hylobates* paternal lineages began around 2.6 (1.9-3.3) mya, involving the divergence of *H. muelleri* lineage from others. Mtgenome data [6] suggested that the first divergence of *Hylobates* maternal lineages occurred relatively earlier around 4.2 (2.5-6.1) mya and the large confidence interval of this time estimate overlaps with the interval of Y chromosome estimate.

Comparatively older mitochondrial and younger Y chromosomal estimates of molecular divergence dates also have been observed in other primates, including African colobine monkeys (the split of *PilicolobusProcolobus*[49]), macaques [50], odd-nosed monkeys [49], chimpanzees [45,51,52] and humans [46,53]. Sex-biased dispersal or other demographic processes have been suggested to explain the differences between mitochondrial and Y chromosomal divergence times in macaques and humans [46,50,53]. For example, Tosi et al. [50] suggested that the relatively recent Y chromosome relative to mitochondrial divergence time estimates in macaques could be explained by sex-biased dispersal (i.e. female philopatry and frequent male dispersal). In gibbons, individuals of both sexes exhibit natal dispersal [54], and although the dispersal age and distance may vary between females and males [55,56], little data exist on the patterns exhibited by most gibbon species. With regard to demographic processes, Tang et al. [53] suggested that an unequal generation length between males and females may contribute to the difference in Y and mtDNA coalescence times in human populations while Wilder et al. [46] suggested the skew in human breeding ratio (e.g. reduced male effective population size due to a polygynous mating system) may explain the more recent time to coalescence of human Y chromosomes. The social and breeding systems of gibbons, although often comprising a breeding pair of adults and their offspring, also features small groups with multiple male or female adults, extrapair copulations, and extra pair paternity [54,57-62].

The complexity and variability of the flexible mating strategies and sex-specific dispersal patterns in gibbons make it difficult to readily elucidate their effects on the divergence time estimates. If the pattern of molecular dating inferred from mtDNA and Y chromosome data is a true reflection of gibbon matrilineal and patrilineal histories, the delayed divergences occurred among paternal lineages might raise speculation of prolonged male-biased gene flow. It has been generally agreed that the genus *Hylobates* originated on the Southeast Asia mainland [6,20,26,27,31]. The two northernmost distributed species (*H. lar* and *H. pileatus*) may have diverged first and then the southern species ( *H. agilisH. klossiiH. moloch* and *H. muelleri*) migrated southward to the Sundaland area [6,26,31]. During the dispersal, the *Hylobates* maternal lineages gradually genetically differentiated from each other and then have diverged into six monophyletic lineages whereas the gene flow mediated through males might have lasted much longer than those through females. This prolonged gene flow may lead to slower fixation of species-specific variation on Y chromosomes, and therefore may result in uncertainty of phylogeographic inferences concerning *Hylobates* based upon Y chromosome data.

Molecular dating makes it possible to estimate the divergence times of populations and species, especially for species without adequate fossil record such as chimpanzees and gibbons [63,64]. Our dating results of Y chromosomes, together with mtDNA estimates [6,20], offer an opportunity to compare the time window of divergence events in gibbon evolution with inferred biogeographical events in the Sundaic region, a topic that is outside the scope of the present study but well described recently [20]. However, we must emphasize that the dates of actual population or species divergences are necessarily more recent than dates of molecular divergences [65,66].

### Monophylies of gibbon genera

The consequence of the relatively low level of sequence variation of the Y chromosome amplicons is apparent in a comparison of the results of phylogenetic analysis of the Y chromosome and mtgenome data. Unlike the strongly supported branching pattern depicted by the mtgenome phylogeny [6], our Y chromosome phylogeny features some weakly supported nodes; however, most of the nodes in the tree were well-supported and provide information relevant to the history of gibbon paternal lineages (Figure 2). Importantly, our Y chromosome tree depicted each of the three sampled genera as monophyletic clades. The consistency of the genus level monophyly inferred from the Y chromosome and mtDNA data [6,20,22,27-31], as well as from the 14 kb-length combined sequences of mtDNA, Y-linked and X-linked loci [26] and the 27.5 kb-length autosomal DNA sequences [34], is concordant with expectations based upon the marked differences among genera in chromosomal numbers and structures [19,67]. Nonetheless, some cases of intergeneric hybridization in captivity have been documented and involve *Hylobates* ×  *Nomascus*[68] and *Hylobates* ×  *Symphalangus*[69]. However, these hybrids were all induced by captive conditions and intergeneric hybrids never have been reported in the wild [68]. Furthermore, the hybrid offspring are expected to be sterile due to the errors of meiotic pairing during gametogenesis [68,69]. Based on the strongly supported genus level monophylies inferred from the Y chromosome (the present study), mtDNA [6,20,30,31,70,71] and autosomal [34] studies, we would suggest that the genetic differentiation at the genus level have been completed in gibbons and the dramatic chromosomal dissimilarity and rearrangements among genera have likely acted as major barriers driving the intergeneric divergences [68].

### Y chromosome and mtDNA phylogenies of *Hylobates* species

Compared to other gibbon genera, *Hylobates* diverged within a relatively short period of time [6,20]. The divergence of the six *Hylobates* species sampled here has been estimated to occur over an interval of only ~1.5 million years [6]. The low sequence variation of Y chromosome loci produced the inferred phylogenetic trees with weak support values on the nodes (Additional file 1). The resolution of trees based on the concatenated dataset showed relatively high resolution compared to the trees of individual amplicons but an unresolved trichotomy within *Hylobates* remains (Figure 2). In our Y chromosome tree, we depict *H. muelleri* as basal species of the genus *Hylobates*, but this placement is inconsistent with other studies which place *H. pileatus*[30,31], *H. moloch*[27] or *H. klossii*[20] as the basal *Hylobates* taxon with strong or weak supports. Although the identity of the basal taxon is still debated, the grouping of four *Hylobates* species with geographic distribution restricted to the Sundaic inlands ( *H. agilisH. klossiiH. moloch* and *H. muelleri*) has been consistently depicted in several studies [6,30,31]. Moreover, as shown in our mtgenome tree of the 26 retrieved sequences (Figure 2), the gibbon phylogeny based on 51 mtgenome sequences recently suggested that the *Hylobates* divergence started with two clades which are further divided into three species pairings: *H. larH. pileatusH. klossiiH. moloch* and *H. agilisH. muelleri*, and thus the absence of a basal species in the *Hylobates* phylogeny [6]. In sum, these results suggest that the sampling of large amounts of sequence variation, as was done using entire mtgenomes, is necessary to resolve divergence events within *Hylobates*. While sequencing the Y chromosome loci benefits the understanding of *Hylobates* patrilineal history, the sequence variation of the loci sampled here was not sufficient for clarification of phylogenetic relationships among these rapidly diverged gibbon species.

### Gene flow between *Hylobates species*

Although *Hylobates* species have been recognized as distinct taxa, three hybrid zones have nonetheless been described within *Hylobates*[23]. These occur between *H. lar* and *H. pileatus* in Thailand, between *H. lar* and *H. agilis* in peninsular Malaysia, and between *H. albibarbis* and *H. muelleri* on Borneo [23]. Of these zones, natural hybridization between *H. lar* and *H. pileatus* has been documented in Khao Yai National Park of Thailand where mating between representatives of the two species or their hybrids and backcross individuals were observed [23,72]. These hybrids were apparently fertile based on identification of the offspring of the first or second generation backcrosses according to pelage and song features [23,72]. Though the currently reported natural hybrid offspring between *H. lar* and *H. pileatus* are limited to the narrow range of the contact zone [72,73], the evidence of these field observations may imply the ongoing gene flow between these two species after their divergence. Moreover, genetic information could be helpful to corroborate this inference of gene flow and genetic data based on the mtDNA and Y chromosome will benefit the detection of possible sex-mediated gene flow [74]. In the present study, we found one of the sampled *H. pileatus* (J16) individuals falling within the *H. lar* clade in our Y chromosome tree, but the mtgenome sequence of this individual was sorted into monophyletic clade of *H. pileatus*[6]. Possible explanations for this paraphyly of *H. pileatus* Y chromosome were misleading inference due to our small sample size or incomplete lineage sorting in the short time since these two species had shared a polymorphic common ancestral population [6]. Alternatively, the individual J16 having a *H. pileatus* mitochondrial genome but a *H. lar* Y chromosome may represent an instance of recent male-mediated gene flow between *H. lar* and *H. pileatus*. Information concerning this individual is extremely limited. Individual J16 was described as *H. pileatus* when was collected in the 1980s and also was judged as a pure *H. pileatus* but not a F1 hybrid by two researchers familiar with gibbon morphological characteristics recently independently from several of its photographs (T. Geissmann and A.R. Mootnick, personal communication). However, the unclear origin and unknown parentage of this captive individual makes it difficult to provide further information about gene flow.

Potential gene flow between gibbon species is not surprising in light of what we know from genetic data about hybridization between closely related lineages in other apes: orangutans, gorillas, bonobos-chimpanzees, Neanderthals-humans, and primates in general [75,76]. Gibbons live in small groups and the social unit consists typically of a breeding pair, and immature presumptive offspring, although groups with multiple adults have been reported [54,57,59,62]. Because both males and females typically leave their natal groups upon reaching maturity and establish new social groups [54], there is the potential for both male and female-mediated gene flow across taxonomic boundaries. The natural hybridizations occurring between *Hylobates* species at the present time [23] motivates future investigation of what role gene flow may play in the evolutionary history of *Hylobates* speciation, and insights are likely to be gained by sequencing of multiple autosomal loci in addition to mtDNA and Y chromosome sequences. Particularly important would be the sampling of individuals of known geographic provenance and subspecies affinity. Although feasible, the sampling of gibbons in the wild is challenging and often limited to the acquisition of fecal samples [20], which tend to produce DNA in low concentration and quality that can impede analyses, though new developments in sequencing technologies can improve the ability to use such samples [77].

## Conclusions

Our study generated a total of over 165 kb sequence data of gibbon Y chromosomes from 26 individuals representing 10 different species. These paternally inherited Y chromosomal sequences confirm the monophyly of the respective gibbon genera as previously suggested using data from maternal mtDNAs, biparental autosomal loci and chromosomal analyses [6,19,20,22,27-31,34,67]. Furthermore, in comparison with mtDNA analyses [6,20,27,30,31], we show different branching patterns in the Y chromosome phylogeny tree (Figure 2) and somewhat more recent estimates of Y-chromosome divergence time (Table 4). Although the results from the small sample sizes of our study limit our interpretations of possible gene flow between *Hylobates* species, we suggest that this gene flow may have occurred recently or be ongoing between closely distributed species, and suggest that the incorporation of autosomal data and a larger sample set is necessary for elucidating any such patterns of gene flow.

## Methods

### Gibbon DNA samples and PCR amplification of Y chromosome loci

All DNA samples used were not acquired specifically for this study and derive from the long-term sample collections of the authors. The samples were originally collected in the course of routine veterinary examinations of captive gibbons. We used high-quality genomic DNA samples from 26 male individuals representing 10 gibbon species, comprising *H. agilis* (n = 3), *H. lar* (n = 10), *H. muelleri* (n = 1), *H. klossii* (n = 1), *H. moloch* (n = 2), *H. pileatus* (n = 2), *S. syndactylus* (n = 3), *N. leucogenys* (n = 1), *N. gabriellae* (n = 2) and *N. concolor* (n = 1) (Additional file 2). An additional eight DNA samples from females were used to test the male-specificity of the primers as described below. We performed whole genome amplification (WGA) on all genomic DNA samples using the multiple displacement amplification procedure implemented in the GenomiPhi HY DNA Amplification Kit (GE Healthcare). The WGA products were purified by ethanol precipitation following manufacturer’s instructions. We quantified the purified WGA products using a NanoDrop spectrophotometer (Thermo Fisher Scientific, Inc.) and used them as templates for subsequent polymerase chain reactions (PCRs) for the amplification of Y chromosomal loci.

From the literature we identified loci on the non-recombining portion of Y chromosome (NRY) and tested 16 published primer pairs (Additional file 2) for male-specific amplification using 10 males (one individual per species), along with eight female individuals (no female individuals were available from *H. klossii* and *N. concolor*). These primers were reported to be capable of amplifying the NRY loci of gibbons or great apes. The primer pairs were considered male-specific when the expected PCR products were obtained in males but not in females. Of the tested primers, seven primer sets were then used to amplify segments from six NRY loci from all male individuals. Sixty or 120 ng of the purified WGA products were used in 50 μl PCR reactions. Detailed information concerning the primer sequences, the PCR conditions and the PCR mix are listed in Additional file 2.

### Sequencing of Y chromosome PCR amplicons

We used the high-throughput 454 sequencing technology with the parallel tagged sequencing approach [78,79] to sequence the seven PCR amplicons from gibbon Y chromosomes following the manufacturer’s instructions (GS FLX platform, Roche). In brief, we pooled the seven purified PCR amplicons by individual in equimolar ratios and then sheared the individual pools by sonication. Individual-specific barcoding adapters were ligated to the DNA fragments in each individual pool so that each of the 26 gibbons had a unique tag. After tagging, the 26 individual pools were mixed together in equimolar ratios and the 454 adapters were ligated to the tagged fragments in the mix pool to produce the sequencing library. We estimated the DNA concentration of this library with quantitative PCR and then sequenced it using the standard GS FLX sequencing procedure. The raw reads were filtered, trimmed and base-called using GS Run Processor application of Genome Sequencer FLX System Software (454 Life Science, Roche). For raw data processing, the 454 read sequence data were sorted according to individual-specific barcode sequences and then classified into the 26 subsets (26 individuals). *De novo* assembly of the reads for each subset was carried out using runAssembly command of GS *De Novo* Assembler 2.0 software (454 Life Science, Roche) to create consensus contigs for each PCR amplicon.

### Sequence data analysis

Homology analyses were applied to the contig sequences of every subset (individual) using BlastN from NCBI (http://www.ncbi.nlm.nih.gov/blast/Blast.cgi). For each individual, seven consensus contigs of the amplicons were entered in BlastN for searching for the sequence matches with high identities under the database Nucleotide collection (nr/nt). All contig sequences showed high similarities to the corresponding homologous sequences of human or other great apes as expected. The Y chromosomal contig sequences were then edited to remove the primer sequences using BioEdit 7.0.5 [80] and the seven edited contig sequences from the same individual were concatenated using DnaSP 5.10.01 [81]. Multiple sequence alignments for each of the amplicon datasets (DAZ-1, DAZ-2, DBY, RPS4Y, SMCY, TSPY and UTY) and for the concatenated dataset were carried out using ClustalW 2.0 [82]. We used DnaSP to calculate two indices of nucleotide diversity: π [83] and θ_w_[84], where π is the average number of nucleotide differences per site between two sequences and θ_w_ is the proportion of number of segregating sites in the sample. All obtained gibbon Y chromosomal sequences have been deposited in Genbank under the accession numbers shown in Additional file 2. For comparison between the nucleotide diversity levels of Y chromosome and mtgenome for gibbons, we retrieved the mtgenome sequences from the same number of 26 individuals to also calculate two indices of π and θ_w_, in which the control regions of these sequences were excluded due to the consideration of the missing data contained [6]. Of these 26 retrieved sequences, 17 individuals are exact same ones used in the Y chromosme dataset and three are the mothers of the individuals T03, T05 and T09, respectively (Figure 2, Additional file 2). Since there is no mtgenome sequence available for the remaining six individuals (23, 103, 1138, 1228, T03 and T08), we used the mtgemone sequences (26, 502, 1135, J10, J12 and J24, shown in Figure 2) of six other individuals which represent the same species. This enabled us to have the same sample sizes for both the Y chromosome and mtgenome datasets used here. GenBank IDs of these mtgenome sequences are: HQ622758, HQ622760-HQ622761, HQ622763-HQ622767, HQ622769, HQ622771, HQ622773- HQ622774, HQ622777-HQ622778, HA622782-HQ622783, HQ622785-HQ622786, HQ622788, HQ622791, HQ622795, HQ622798, HQ622802, HQ622806-HQ622808.

### Phylogenetic analyses

The homologous Y chromosomal sequences of three primate species (human, chimpanzee and macaque) were used as out groups (Additional file 2) for the following phylogenetic reconstructions. We aligned the out group sequences with the datasets of the individual amplicons separately as well as the concatenated dataset. The variable, invariable and parsimony-informative sites in these alignments were identified using DnaSP. We reconstructed Y chromosome phylogenies of gibbons using two different approaches, maximum likelihood (ML) and Bayesian inference (BI). We generated partitioned BI and ML analyses by separating the concatenated dataset into seven partitions (seven PCR amplicons), while non-partitioned BI and ML analyses were applied to the individual-locus datasets. Non-partitioned and partitioned ML analyses were performed using RAxML 7.2.8 [85,86] with independent GTR (general time reversible) substitution models applied to seven datasets/partitions. We conducted non-partitioned and partitioned BI analyses using MrBayes 3.1.2 [87]. The best-fit nucleotide substitution models were assessed using the Akaike information criterion (AIC) by Model-Generator 0.85 [88]. The GTR + I model was used for the datasets/partitions of DAZ-1 and TSPY, GTR + Γ for DBY, GTR for DAZ-2 and SMCY, and HKY for RPS4Y and UTY. Four Metropolis-coupled Markov chain Monte Carlo (MCMC) analyses were run twice for 1 × 10^6^ generations for individual-locus datasets and for 5 × 10^6^ generations for the concatenated dataset and sampled every 100 generations with a burn-in of 25%. For comparison between Y chromosome and mtgenome phylogenies, we used the 26 retrieved mtgenome sequences (as used in the calculation of nucleotide diversity levels) to reconstruct mtgenome phylogeny tree using partitioned BI and ML analyses as described in Chan et al., in which the sequences of 22 tRNA genes, 2 rRNA genes and 13 protein-coding genes were used for tree reconstructions [6].

### Estimation of divergence times

We estimated divergence times in the gibbon Y chromosome phylogeny using a Bayesian approach implemented in the program BEAST 1.6.1 [89]. We used two fossil-based calibration points as normal priors to obtain the posterior distribution of the estimated divergence times: the split of hominoids-cercopithecoids (~26.5 mya ± 2.5 mya, [90,91]) for the node *Macaca*-apes, and the divergence between *Homo* and *Pan* (~6.5 mya ± 0.5 mya, [92-94]) for the human-chimpanzee node. The concatenated dataset was used and divided into seven partitions as described above for the BEAST analysis. Different substitution models were assigned to the partitions as described in the phylogenetic partitioned BI analysis. Two independent BEAST runs were carried out using uncorrelated lognormal relaxed clock model [95] without an *a priori* fixed reference topology, along with following settings: Yule speciation process in tree prior, 4 × 10^7^ generations of MCMC steps, and sampling every 4000 generations. Convergence was assessed in Tracer 1.5 [96] after excluding the first 8 × 10^6^ generations as burn-in. We combined the log output files from two individual BEAST runs using LogCombiner 1.6.1 [89]. The effective sample size (ESS) values exceeded 550 for all parameters. The combined tree log files were analyzed using TreeAnnotator 1.6.1 [89] to calculate the maximum-clade-credibility topology and mean node heights from the posterior distribution of the trees. We visualized the tree results using FigTree 1.3.1 [97].

## Abbreviations

mtDNA, Mitochondrial DNA; mtgenome, Mitochondrial genome; mya, Million years ago; WGA, Whole genome amplification; PCR, Polymerase chain reaction; NRY, Non-recombining portion of Y chromosome; ML, Maximum likelihood; BI, Bayesian inference; GTR, General time reversible; AIC, Akaike information criterion; MCMC, Markov chain Monte Carlo; ESS, Effective sample size; HPD, Highest posterior density; TMRCA, Time to most common ancestor.

## Competing interests

The authors declare that they have no competing interests.

## Authors’ contributions

Y-CC carried out the experimental work of the study, analyzed the data and wrote the manuscript. CR contributed samples and wrote manuscript. MI-M and EI contributed samples and helped to draft the manuscript, and C-CS contributed samples. LV conceived of the study and wrote the manuscript. All authors read and approved the final manuscript.

## Supplementary Material

Additional file 1 **Supplementary Figure S1.** Phylogeny trees of individual Y chromosomal amplicons. Click here for file

Additional file 2 **Supplementary Tables S1 to S4. **This file includes the information of gibbon samples, primer sequences and PCR conditions used in the present study. The list of GenBank IDs for outgroup sequences and newly obtained sequences of gibbons is also included. Click here for file
