# Supplementary material for: A comparative analysis of Y chromosome and mtDNA phylogenies of the Hylobates gibbons
Source: BMC Evol Biol. 2012 Aug 21;12:150. doi: 10.1186/1471-2148-12-150 (PMC3444420; doi:10.1186/1471-2148-12-150)

Figure S1 Phylogeny trees of individual Y chromosomal amplicons:
Maximum likelihood (ML) and Bayesian (BI) majority-rule trees of gibbon phylogenies based on the sequences from different Y chromosomal amplicons: DAZ-1, DAZ-2, DBY, RPS4Y, SMCY, TSPY and UTY. The ML trees are on the left and Bayesian trees on the right.

ML and BI trees of DAZ-1

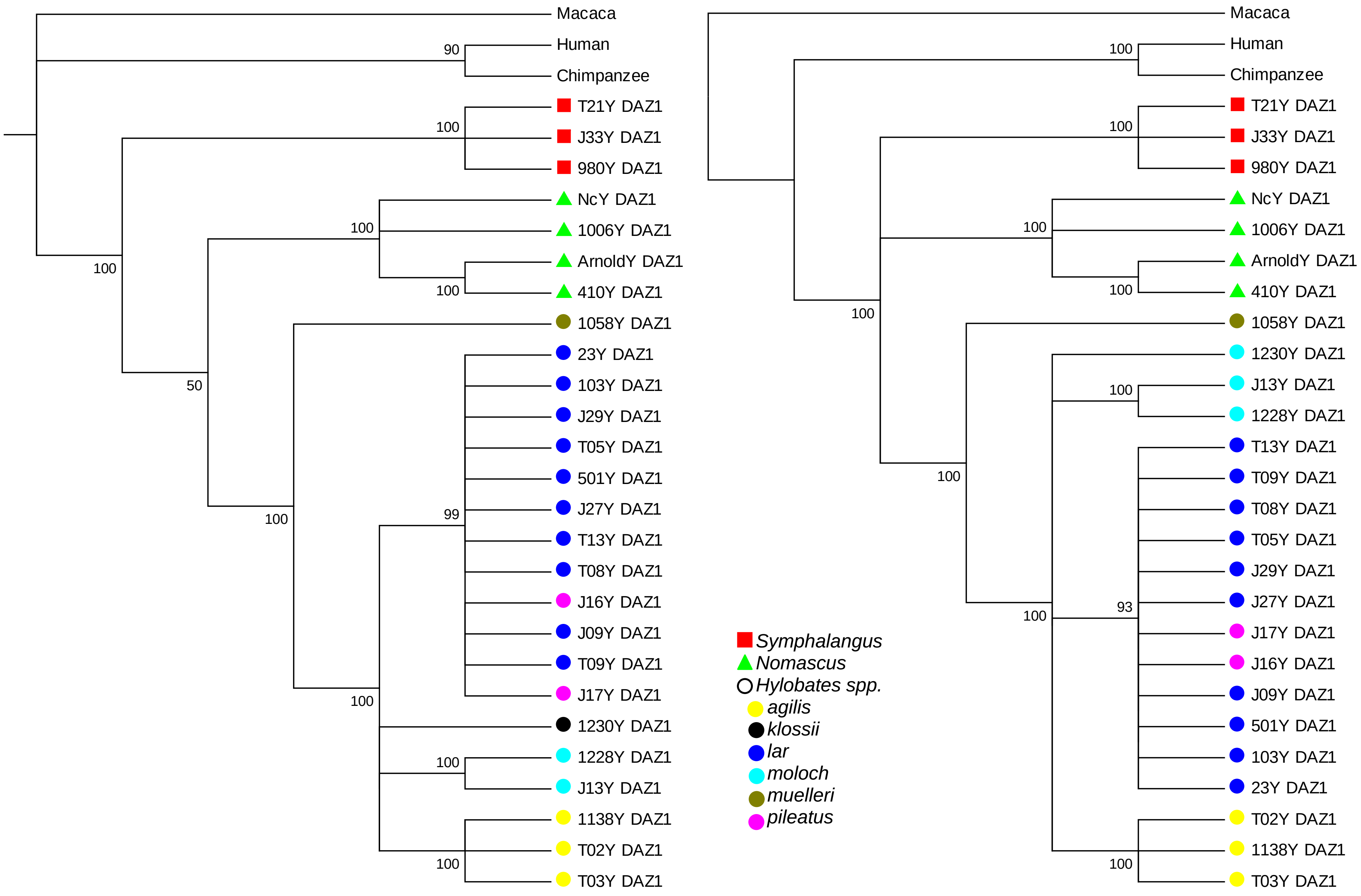

ML and BI trees of DAZ-2

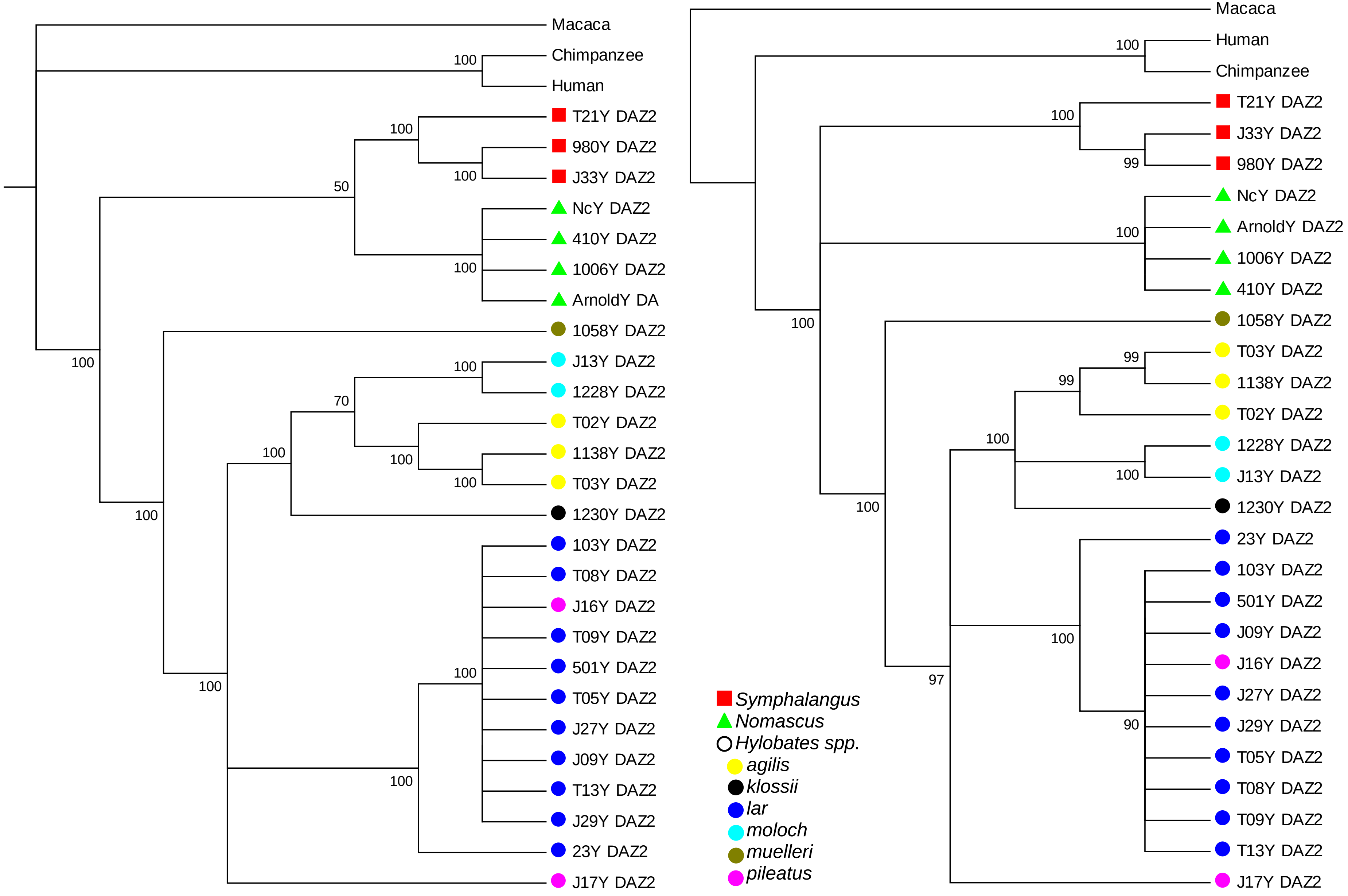

ML and BI trees of DBY

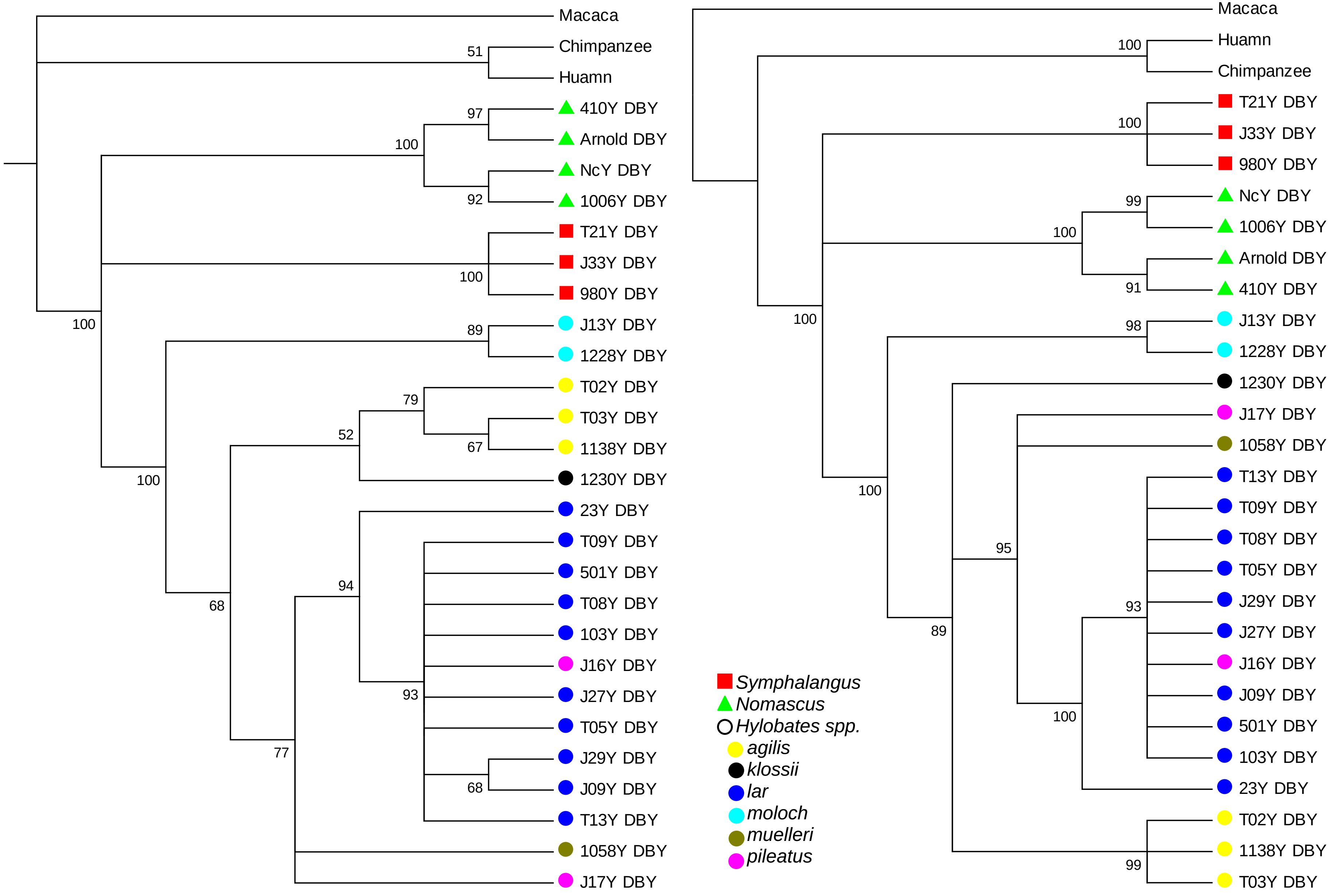

ML and BI trees of RPS4Y

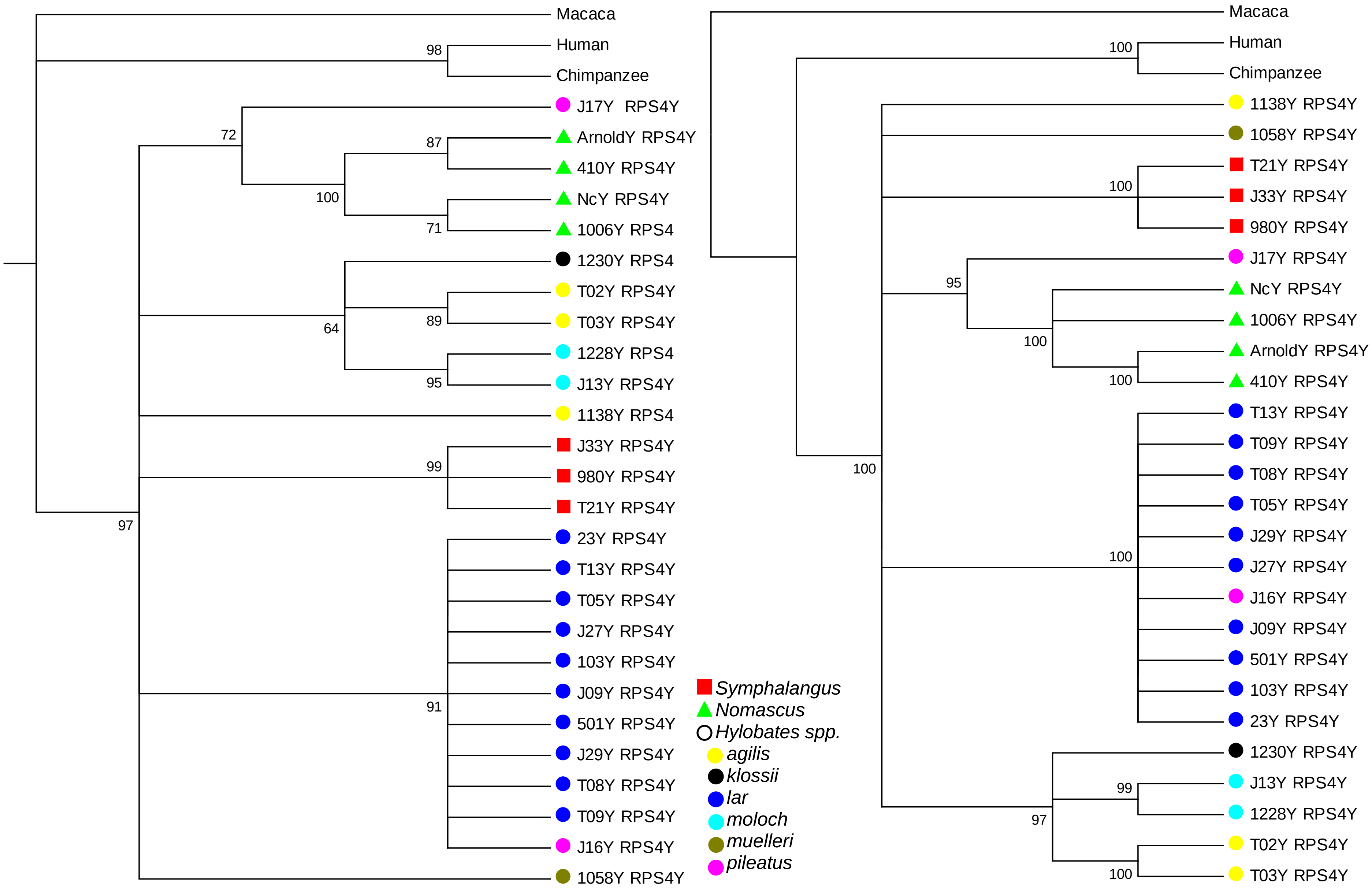

ML and BI trees of SMCY

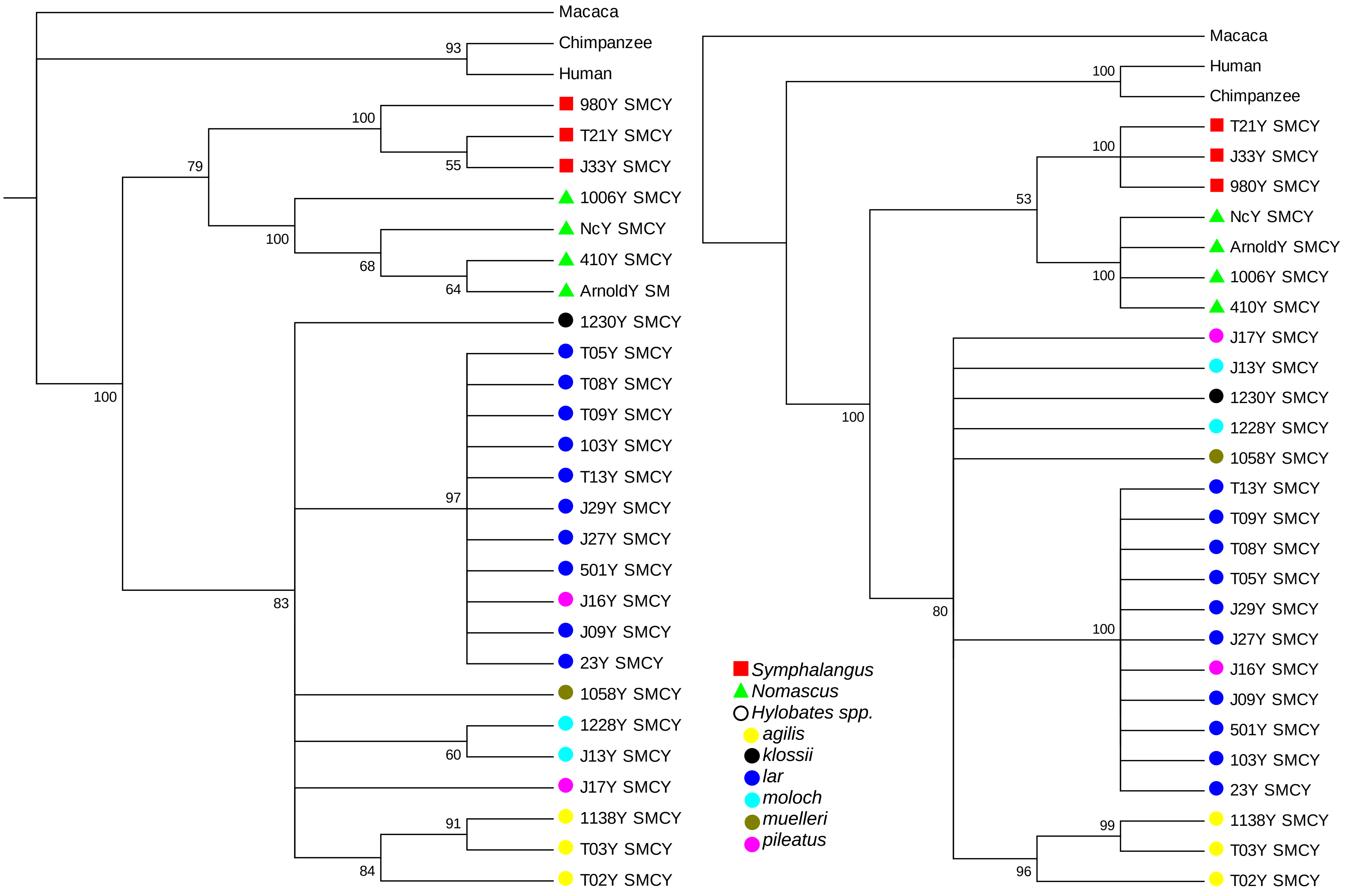

ML and BI trees of TSPY

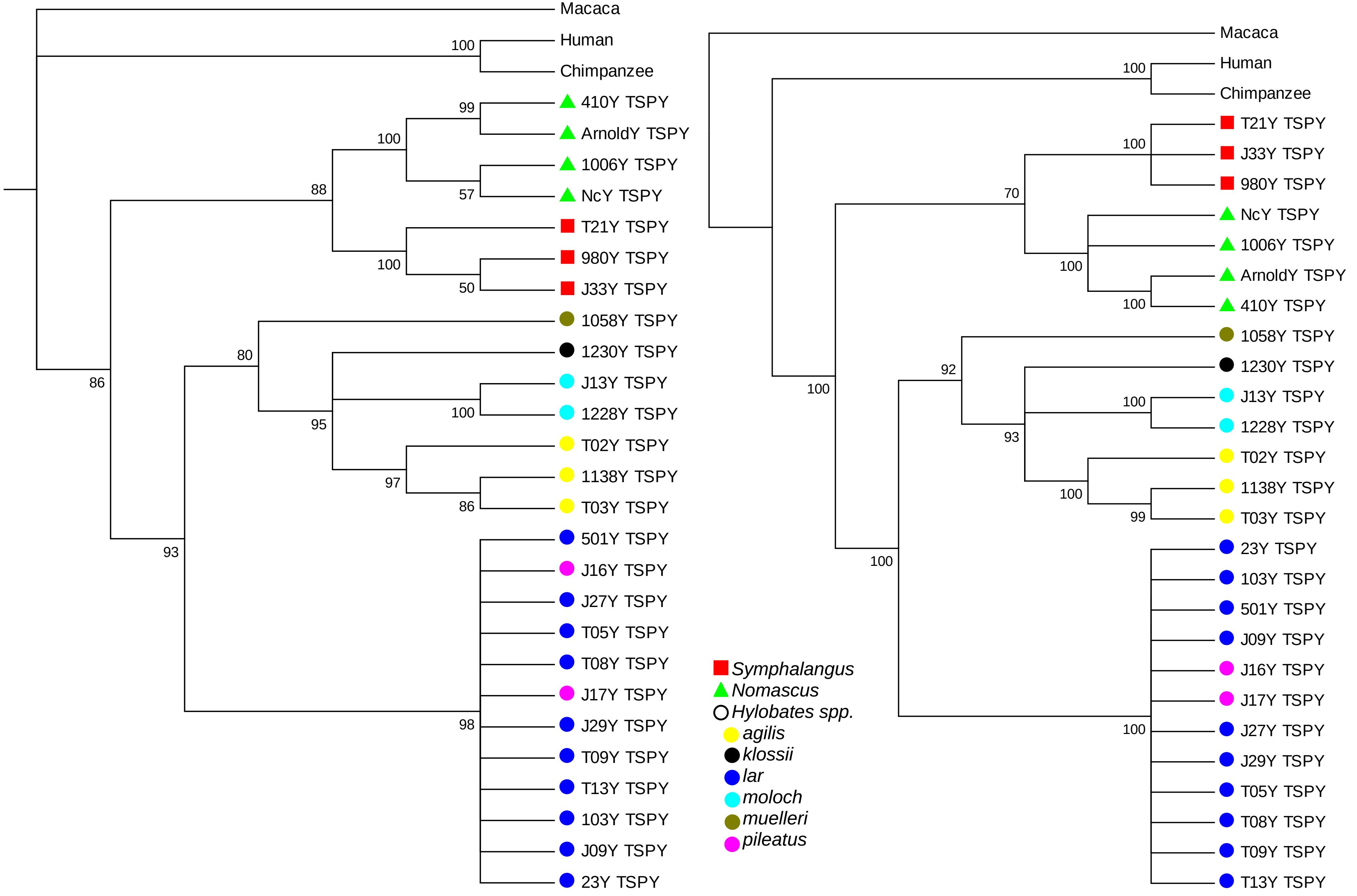

ML and BI trees of UTY

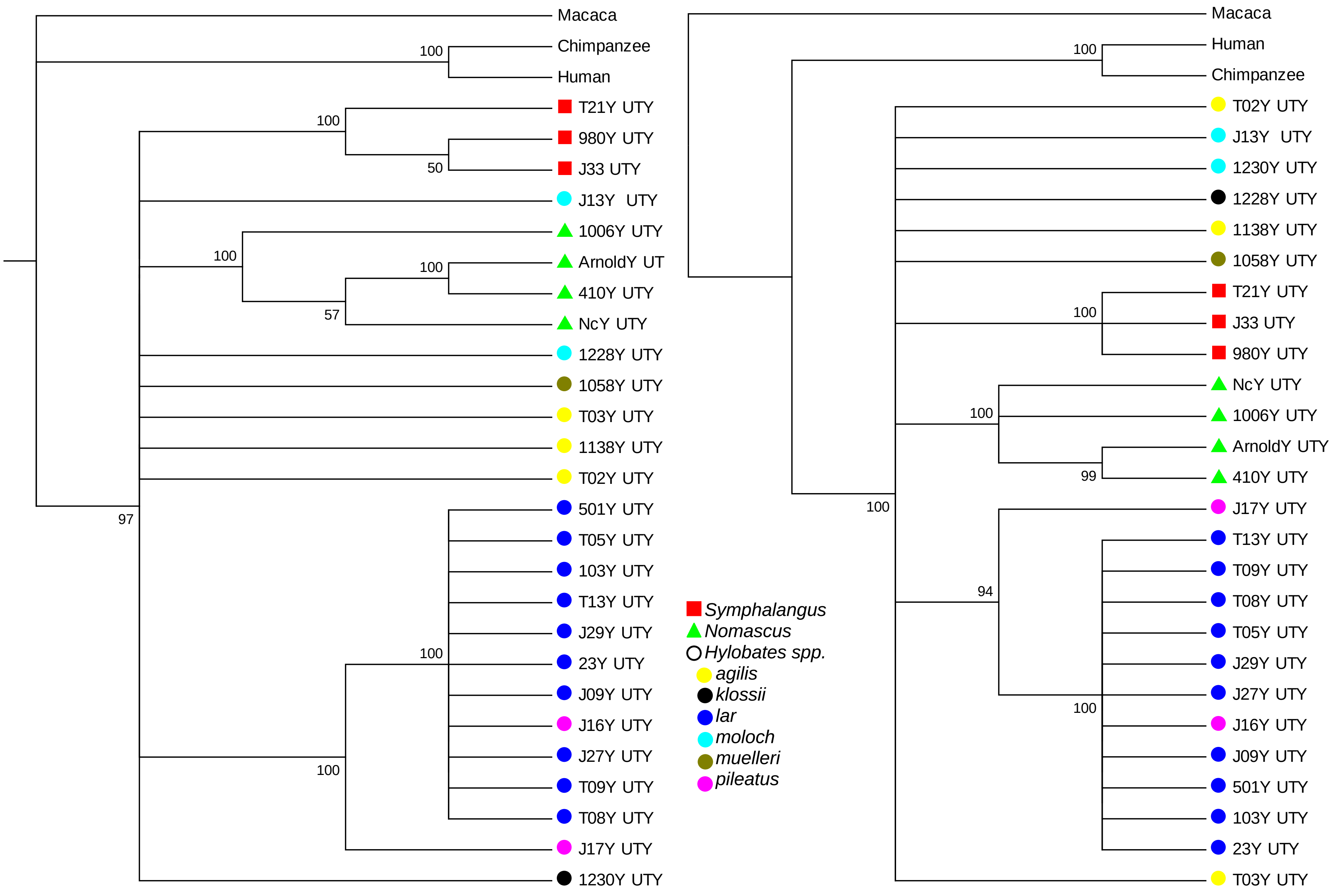

Supplement: Additional file 1 — Supplementary Figure S1. Phylogeny trees of individual Y chromosomal amplicons. [file 1471-2148-12-150-S1.pdf]
