# Supplementary material for: A comparative analysis of Y chromosome and mtDNA phylogenies of the Hylobates gibbons
Source: BMC Evol Biol. 2012 Aug 21;12:150. doi: 10.1186/1471-2148-12-150 (PMC3444420; doi:10.1186/1471-2148-12-150)
Supplement: Additional file 2 — Supplementary Tables S1 to S4. This file includes the information of gibbon samples, primer sequences and PCR conditions used in the present study. The list of GenBank IDs for outgroup sequences and newly obtained sequences of gibbons is also included. [file 1471-2148-12-150-S2.pdf]

Supplementary Materials

Table S1. Gibbon samples used in the present study

Genus	Species	onwer's ID/Barcode ID	Working ID [b]	Origin[a]	Current deposition[a]
Hylobates	*agilis*	20050293D10	T02	Taipei Zoo	Taipei Zoo
		20060466D10	T03	Taipei Zoo	Taipei Zoo
		1138	1138	Bristol Zoo	German Primate Center
	lar	20040083D10	T05	Taipei Zoo	Taipei Zoo
		20040111D10	T08	Taipei Zoo	Taipei Zoo
		20040112D10	T09	Taipei Zoo	Taipei Zoo
		20040286D10	T13	Taipei Zoo	Taipei Zoo
		23	23	Wuppertal Zoo	German Primate Center
		103	103	Nuremberg Zoo	German Primate Center
		501	501	Nuremberg Zoo	German Primate Center
		2356	J09	Dusit Zoo	WRC, Kyoto University
		1982	J27	Chiang Mai Zoo	WRC, Kyoto University
		3400	J29	PRI, Kyoto University	WRC, Kyoto University
	muelleri	1058	1058	Rostock Zoo	German Primate Center
	klossii	1230	1230	Twycross Zoo	German Primate Center
	moloch	2488	J13	Ragunan Zoo	WRC, Kyoto University
		1228	1228	Twycross Zoo	German Primate Center
	pileatus	2360	J16	Dusit Zoo	WRC, Kyoto University
		2361	J17	Dusit Zoo	WRC, Kyoto University
Symphalangus	*syndactylus*	20060592D10	T21	Taipei Zoo	Taipei Zoo
		980	980	Krefeld Zoo	German Primate Center
		1973	J33	Padang, Sumatra, Indonesia	WRC, Kyoto University
Nomascus	*leucogenys*	1006	1006	Duisburg Zoo	German Primate Center
	gabriellae	Arnold	Arnold	Leipzig Zoo	MPI-EVA
		Falco	Falco	Leipzig Zoo	MPI-EVA
	concolor	1231	Nc	Twycross Zoo	German Primate Center

[a] Abbreviations: PRI, Primate Research Institute; WRC, Wildlife Research Center; MPI-EVA, Max-Planck Institute for Evolutionary Anthropology

[b] The mtgenome sequences of the individuals underlined are retrieved for the reconstruction of mtgenome phylogeny, in which individuals T02, T05 and T09 are the sons of individuals T01, T05 and T10, respectively, as shown in Figure 2.

Table S2. List of primers tested in this study for the amplification of the non-recombining portion of Y chromosome

NCBI name	Gene name*	primer pair	Reference	Note
DAZ1	DAZ	1990FY x 3760RYsiam	Makova and Li, 2002 [1]	Applied to all male individuals
		6996FY x 8754RY	Makova and Li, 2002 [1]	Failed to amplify in every species
		18219FY x 19599RY	Makova and Li, 2002 [1]	Applied to all male individuals
		18264F x 21252RY	Makova and Li, 2002 [1]	Amplification occurred in both sexes
		2384FY x 4809RY	Makova and Li, 2002 [1]	Failed to amplify in every species
		25321FY x 27521RY	Makova and Li, 2002 [1]	Failed to amplify in every species
DDX3Y	DBY	DBY1-F x DBY1-R	Hellborg and Ellegren, 2003 [2]	Failed to amplify in every species
		DBY5-F x DBY5-R	Hellborg and Ellegren, 2003 [2]	Applied to all male individuals
		DBY6-F x DBY6-R	Hellborg and Ellegren, 2003 [2]	Failed to amplify in every species
EIF1AY	EIF1AY	EI-1 x EI-2	Hong et al., 2007 [3]	Failed to amplify in every species
RPS4Y1	RPS4Y	RPS4Y4-F x RPS4Y4-R	Hellborg and Ellegren, 2003 [2]	Applied to all male individuals
		RPS4Y-F x RPS4Y-R	Samollow et al., 1996 [4]	Failed to amplify in every species
KDM5D	SMCY	SMCY7-F x SMCY7-R	Hellborg and Ellegren, 2003 [2]	Applied to all male individuals
		SMCY11-F x SMCY11-R	Hellborg and Ellegren, 2003 [2]	Failed to amplify in every species
TSPY	TSPY	TSPY-A x TSPY-B	Kim et al., 1996 [5]	Applied to all male individuals
UTY	UTY	UTY11-F x UTY11-R	Hellborg and Ellegren, 2003 [2]	Applied to all male individuals

*Gene name aliases used in the references and this study.

Table S3. Primer sequences, sources of primers, and PCR conditions used in this study

Locus	Primer name	Primer sequence (5'-3')	PCR condition	Estimated size (bp)	PCR Master Mix*
DAZ	1990FY (DAZ-1F)	F: TGGGGAAACATAAGGGAGAAAAAAGTA	92°C 2 min, (92°C 10 sec, 57°C 15 sec, 68°C 4 min) × 9 cycles, (92°C 10 sec, 57°C 15 sec, 68°C 4 min) × 21 cycles with 20 sec extension/cycle, 68°C 7 min	1350	A
	3760RYsiam (DAZ-1R)	R: GGCTTGGCACGGAAAGACG			
DAZ	18219FY (DAZ-2F)	F: GCTTAATCCAAACAATCAGTGAGACA	92°C 2 min, (92°C 10 sec, 57°C 15 sec, 68°C 4 min) × 9 cycles, (92°C 10 sec, 57°C 15 sec, 68°C 4 min) × 21 cycles with 20 sec extension/ cycle, 68°C 7 min	1600	A
	19599RY (DAZ-2R)	R: GCCTTAACTTTAAGAGAATATTGGGGA			
DBY	DBY5-F	F: CTTTGAGAAATATGATGATATA	95°C 10 min, touchdown with an annealing temperature of 55°-45°C over 20 cycles, (95°C 1 min, 45°C 1 min, 72°C 1 min 30 sec) × 30 cycles, 72°C 10 min	750	B
	DBY5-R	R: CGAGTAAGTTCAATGTTC			
RPS4Y	RPS4Y4-F	F: GAACAGATTCTCTTCCGTCG	95°C 10 min, touchdown with an annealing temperature of 63°-53°C over 20 cycles, (95°C 1 min, 53°C 1 min, 72°C 1 min 30 sec) × 30 cycles, 72°C 10 min	600	B
	RPS4Y4-R	R: GCTGCAACACGCTTTAAGTG			
SMCY	SMCY7-F	F: TGGAGGTGCCCRAARTGTA	95°C 10 min, touchdown with an annealing temperature of 63°-53°C over 20 cycles, (95°C 1 min, 53°C 1 min, 72°C 1 min 30 sec) × 30 cycles, 72°C 10 min	500	B
	SMCY7-R	R: AACTCTGCAAASTRTACTCCT			
TSPY	TSPY-A	F: AGCCAGGAAGGCCTTTTCTCG	94°C 4 min, (94°C 1 min, 60°C 1 min, 72°C 1 min 30 sec) × 30 cycles, 72°C 7 min	780	B
	TSPY-B	R: CCATGTAGCTCAGCATGTCTTCAT			
UTY	UTY11-F	F: CATCAATTTTGTAYMAATCCAAAA	95°C 10 min, touchdown with an annealing temperature of 63°-53°C over 20 cycles, (95°C 1 min, 53°C 1 min, 72°C 1 min 30 sec) × 30 cycles, 72°C 10 min	900	B
	UTY11-R	R: TGGTAGAGAAAAGTCCAAGA			

*The final concentration of each reagent in a PCR Master Mix of 50 ul reaction volume. A, a PCR Master Mix consists of 100 ng of the purified WGA products, 0.3 µM of each forward and reverse primer, 500 µM of each dNTP, and 3.5 units of the Expand Long Range Enzyme mix (Roche) with 1X Expand Long Range Buffer containing $MgCl_2$. B, a PCR Master Mix consists of 100 ng of the purified WGA products, 2.2 mM $MgCl_2$, 0.2 µM of each forward and reverse primer, 200 µM of each dNTP, and 2 units of AmpliTaq Gold DNA polymerase (Applied Biosystems, Roche) with 1X AmpliTaq Gold PCR buffer II.

Table S4. GenBank IDs for outgroup sequences and newly obtained sequences of gibbons in this study

Amplicons	*Homo sapiens*	*Pan troglodytes*	*Macaca mulatta*	Gibbons
DAZ-1	AF483577	AC147143 (62937-64614)	AC217138 (113167-114796)	JN871346-JN871371
DAZ-2	AF483578	AC147143 (51140-52443)	AC217138 (98953-100288)	JN871320-JN871345
DBY	NG_012831 (13912-14646)	NC_006492 (18025162-18025912)	AC213321 (74334-75043)	JN871424-JN871449
RPS4Y	AF041427 (19681-20250)	NC_006492 (23753790-23754359)	AC217136 (84539-85106)	JN871398-JN871423
SMCY	AF273841 (45121-45592)	AF440161	AC215644 (72205-71739)	JN871476-JN871501
TSPY	M98524 (1158-1895)	Kim and Takenaka 1996*	AF425276	JN871372-JN871397
UTY	AF265575	NC_006492 (18457945-18458832)	AC212004 (1240-2129)	JN871450-JN871475

The numbers in parenthesis next to the GenBank accession numbers indicate the regions used as outgroup sequences in the phylogenetic analyses. * The TSPY sequence of *Pan troglodytes* is shown in Kim and Takenaka (1996) [6].

References

1. Makova KD, Li W-H: **Strong male-driven evolution of DNA sequences in humans and apes.** *Nature* 2002, **416:**624-626.
2. Hellborg L, Ellegren H: **Y chromosome conserved anchored tagged sequences (YCATS) for the analysis of mammalian male-specific DNA.** *Mol Ecol* 2003, **12:**283-291.
3. Hong KW, Huh JW, Kim DS, Ha HS, Kim HS: **Molecular relationship of hylobates based on Alu elements of the Y chromosome.** *Korean J Genet* 2007, **29:**379-387.
4. Samollow PB, Cherry LM, Witte SM, Rogers J: **Interspecific variation at the Y-linked RPS4Y locus in hominoids: implications for phylogeny.** *Am J Phys Anthropol* 1996, **101:**333-343.
5. Kim HS, Hirai H, Takenaka O: **Molecular features of the TSPY gene of gibbons and Old World monkeys.** *Chromosome Res* 1996, **4:**500-506.
6. Kim HS, Takenaka O: **A comparison of TSPY genes from Y-chromosomal DNA of the great apes and humans: sequence, evolution, and phylogeny.** *Am J Phys Anthropol* 1996, **100:**301-309.
